# Comment on “Quantum interference effects in biphenyl dithiol for gas detection” by J. Prasongkit and A. R. Rocha, *RSC Adv.*, 2016, **64**, 59299–59304

**DOI:** 10.1039/c9ra00451c

**Published:** 2020-01-10

**Authors:** Anton Grigoriev, Hassan Jafri, Klaus Leifer

**Affiliations:** Condensed Matter Theory Group, Division of Material Theory, Department of Physics and Astronomy, Uppsala University Box 516 SE-751 20 Uppsala Sweden; Group of Electron Microscopy and Nano-Engineering, Applied Materials Science, Department of Engineering Sciences, Uppsala University Box 534 SE-751 21 Uppsala Sweden klaus.leifer@angstrom.uu.se

## Abstract

The paper [Prasongkit *et al.*, *RSC Adv.*, 2016, **64**, 59299] by Prasongkit and Rocha calculates the binding energy of gas molecules attached to 1-8-biphenyl-dithiol (BPDT) molecules. We find from our calculations, that the binding energies calculated for the NO_2_ molecules are too low, most likely due to lacking optimization of the site at which the gas molecule binds to the BPDT. Though not shown explicitly here, the same statement might apply to the other gas molecules used in this paper.

## Abstract 2

We find from our calculations that the binding energies for the NO_2_ molecules, calculated in this paper, are too low, most likely due to the lacking optimization of the site at which the gas molecule binds to the BPDT.

## 1 Comment

In this comment to Prasongkit's *et al.* paper ref. 1, we criticize the scientific results of this paper. Prasongkit *et al.* obtain very low binding energies of 0.09–0.17 eV between the gas molecules (NO_*x*_, CO and NH_3_) and the BPDT molecules. These energies are so low that it is doubtful if the gas molecule–BPDT bond would withstand thermal vibration energies. In fact, our calculations could show that the variation and energy optimization of the binding site of the gas molecule, NO_2_ in this case to the BPDT is between 1.04 eV and 2.04 eV large. As a consequence, other conclusions based on the very low binding energies in the Prasongkit papers such as changes in conductance seem unrealistic. Thus, we have the strong suspicion that the binding site in the Prasongkit paper was not accurately optimized and we comment on this in the following.

To understand, why the underlying binding mechanism is important for molecular sensing, calculations were carried out from first principles with a method based on density functional theory (DFT) as implemented in the Siesta package.^2^ The relaxed molecular structures were inserted as their dithiolates between two reconstructed Au(100) surfaces and relaxed once more to optimize the Au–S bonding. The device consists of three parts: left electrode, molecule, and right electrode. The electrodes are modeled by six layers of gold atoms where the three outer layers are relaxed, while the others are kept at the experimental bulk positions. In the lateral dimension a 3 × 3 supercell (8.95 Å × 12.66 Å) was used, large enough to remove interactions between periodic images. All relaxations are performed at the DFT level with the SIESTA package and core electrons are modeled using Troullier–Martins^3^ soft norm-conserving pseudopotentials, with the mesh cut off was 300 eV and Brillouin zone integration for the supercell was sampled by 3 × 3 × 1 *k*-points. The valence electrons are expanded in a basis set of local orbitals using a double-*ζ* plus polarization orbital (DZP) set for electrons in the molecule and a single-*ζ* plus polarization orbital (SZP) for electrons in the gold electrode. The GGA was used for the exchange-correlation functional.^4^

**Fig. 1:**
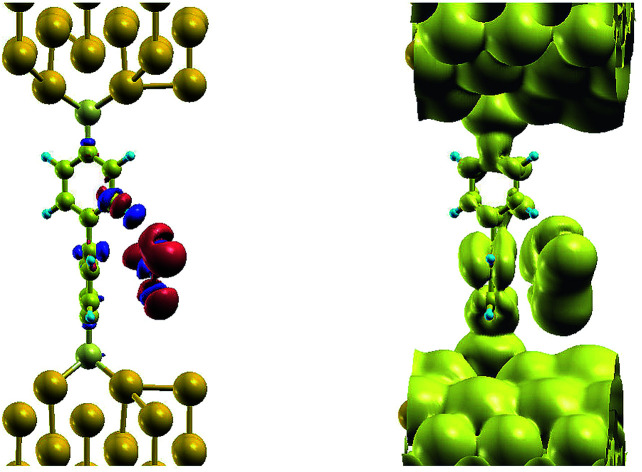
Isosurface plots of charge density difference for NO_2_ adsorption on BPDT (left) and LDOS (±0.1 eV around the Fermi level, right).

The effect of the radical molecule, such as NO_2_, on electron transport between two closely spaced gold electrode would be in introduction of electronic states close to Fermi level of metal electrodes, that would enable resonance tunneling between them. However, there is no mechanism that binds the gas molecule, neither is the size of NO_2_ sufficient to bridge the gap in space between the electrodes, spaced to fit BPDT molecule in between. Introducing BPDT as a sensor we solve the problem of temporarily binding BPDT: 1 shows the charge density difference for NO_2_ adsorption, that demonstrates clear, yet weak hydrogen bonding (blue deficiency blob around one of one of the 4 protons of the upper benzene ring of the molecule), while the body of the gas molecule is accommodated at the face of the lower ring with vdW interaction. In turn, this position of the gas introduces a sufficient distortion on the nearest benzene ring, which makes it possible for the metal-induced gap states (MIGS) induced from the gold to penetrate much further along BPDT, as can be seen on a LDOS plot nearby. Density of states, that would otherwise penetrate across sulfur and up to a first carbon atom in the ring, as seen on a top benzene ring, now resides on the whole lower ring, providing a conduction path for tunneling electrons. In other words, the real space span between the electrodes, that otherwise would be occupied by the MIGS in the HOMO–LUMO gap of the BPDT has now shrank by a half, facilitating electron tunneling at low bias (Fig. 1).

## Conflicts of interest

The group of K. Leifer working in Electron Microscopy and Nano-Engineering at Uppsala University, Sweden, has carried out gas sensor measurements since 2009 starting with measurements on graphene.^5^ On the background of the gas sensing work on graphene, Leifer proposed in 2011 to build a nanoMoED (nanomolecular electronics) device functionalized with BPDT and try to carry out gas sensing experiments with environmental gases. This idea was nourished by the interdisciplinary centre at Uppsala University, U3MEC (Uppsala University Unimolecular Electronics Centre).

After successful initial results, in 2013, we gathered again with the colleagues from theory to discuss possible DFT calculations of the gas sensing effects in uni-molecular devices. At this time, J. Prasongkit was a guest researcher at Uppsala University and our theory colleagues invited her to the meetings.

## Supplementary Material
